# Abdominal Caesarian Section

**Published:** 1913-10

**Authors:** 


ABDOMINAL CAESARIAN SECTION. Josue A. Beruti, *Revista del Circulo Medico Argentina.* Beruti reports a case of an Italian woman, 30 years old, on whom this operation was performed four times. As a child she had rickets and suffered with a great deformity of the bones which finally resulted in a double spontaneous subluxation of the femora. Three of the incisions were in the same line—slight thinning of the abdominal wall resulting, but no hernia. A very slight thinning of the uterine wall was noted. No untoward symptoms accompanied any of the deliveries. Her four children, all boys, are alive and well. This case is reminiscent of one which occurred in Boston some years ago. This woman had had three Cæsarian Sections. The fourth labor set in and surgical assistance was summoned hurriedly. But before the surgeons arrived the child was born per vias naturas, which proves at least that the musculature of the uterus had not been weakened to any great extent by previous operative measures.

